# A giant mass in the neck of a young men: a case of anaplastic thyroid cancer

**DOI:** 10.11604/pamj.2018.30.151.15156

**Published:** 2018-06-20

**Authors:** Carlos Eduardo Salazar-Mejía, Luis Alberto Pérez-Arredondo

**Affiliations:** 1 Universidad Autonoma de Nuevo Leon, Facultad de Medicina y Hospital Universitario “Dr. Jose Eleuterio Gonzalez”, Oncology Service, Department of Internal Medicine, Monterrey, Nuevo Leon, Mexico

**Keywords:** Anaplastic Thyroid Cancer, Metastatic Cancer, Young men

## Image in medicine

A 29-year-old man presented with a 4-month history of a mass in the left lateral region of the neck that was painful to palpation. Growth of this mass caused a change in his tone of voice, dysphagia to solids that progressed to liquids, and dyspnea. He also referred a weight loss of 3 kg. He had no previous medical history. Physical examination of the neck showed a left ganglionar conglomerate, which covered all areas of the neck and the supraclavicular hollow and that extended to the opposite side with limitation of cervical movement. There was involvement of the left cranial nerves XI and XII. Laboratory tests revealed a TSH serum level of 5.19 uU/ml (0.27-4.2 uU/mL), a free T4 level of 0.80 ng% (0.93-1.7 ng%), and a thyroglobulin of 12.2 ng/ml (1.40-78.0 ng/ml). Histopathological analysis showed an anaplastic carcinoma of the thyroid. Due to the great extension of the disease and metastasis to the lung demonstrated by tomography, it was decided to initiate a chemotherapy regimen based on cisplatin and doxorubicin. Despite this treatment, the patient died three months later. Anaplastic thyroid cancers are extremely aggressive undifferentiated tumors with rapid progression and very poor outcome with a median overall survival of less than six months. For unresectable disease in patients who desire active therapy, radiation and/or chemotherapy is the preferred option for local control of the disease. Palliative care is important in this setting and should be added to any treatment plan.

**Figure 1:**
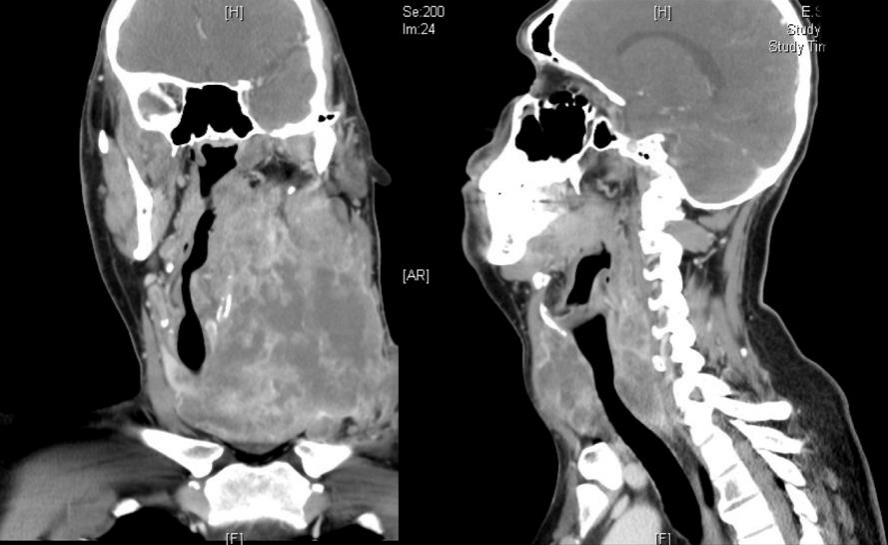
A CT scan showing a large left cervical mass displacing the trachea and vascular structures of the neck

